# Long-term outcomes and potential mechanisms of offspring exposed to intrauterine hyperglycemia

**DOI:** 10.3389/fnut.2023.1067282

**Published:** 2023-05-15

**Authors:** Yi-Shang Yan, Chun Feng, Dan-Qing Yu, Shen Tian, Yin Zhou, Yi-Ting Huang, Yi-Ting Cai, Jian Chen, Miao-Miao Zhu, Min Jin

**Affiliations:** ^1^Department of Reproductive Medicine, The Second Affiliated Hospital, School of Medicine, Zhejiang University, Hangzhou, China; ^2^Key Laboratory of Reproductive Genetics, Ministry of Education, School of Medicine, Zhejiang University, Hangzhou, China; ^3^Department of Operating Theatre, The First Affiliated Hospital of Wenzhou Medical University, Wenzhou, China

**Keywords:** maternal diabetes, offspring, fetal development, long-term outcomes, potential mechanisms

## Abstract

Diabetes mellitus during pregnancy, which can be classified into pregestational diabetes and gestational diabetes, has become much more prevalent worldwide. Maternal diabetes fosters an intrauterine abnormal environment for fetus, which not only influences pregnancy outcomes, but also leads to fetal anomaly and development of diseases in later life, such as metabolic and cardiovascular diseases, neuropsychiatric outcomes, reproduction malformation, and immune dysfunction. The underlying mechanisms are comprehensive and ambiguous, which mainly focus on microbiota, inflammation, reactive oxygen species, cell viability, and epigenetics. This review concluded with the influence of intrauterine hyperglycemia on fetal structure development and organ function on later life and outlined potential mechanisms that underpin the development of diseases in adulthood. Maternal diabetes leaves an effect that continues generations after generations through gametes, thus more attention should be paid to the prevention and treatment of diabetes to rescue the pathological attacks of maternal diabetes from the offspring.

## 1. Introduction

Maternal diabetes mellitus, defined as glucose intolerance during pregnancy, can be classified into gestational diabetes mellitus (GDM) and pregestational diabetes mellitus (PGDM) (1, 2). The former is diagnosed when hyperglycemia is first detected during pregnancy and often returns to a normal glucose level after delivery (3). The latter condition happens when diabetic women, often with type 1 or type 2 diabetes mellitus (T1DM or T2DM), get pregnant (4). Intrauterine hyperglycemia may not only lead to adverse outcomes in pregnant patients and fetuses, such as preeclampsia (5, 6) and macrosomia (7, 8), but may also leave an impact on offspring in the long term (9). According to the theory of Developmental Origins of Health and Disease (10) and the thrifty phenotype hypothesis (11), a poor maternal condition and an intrauterine abnormal environment reprogram the fetus to a metabolic pattern that is adaptive to an insufficient nutrition (12). After birth, the thrifty phenotype lasts and encounters relatively abundant nutrients, and the body functions present a catch-up mode that leads to an eventually overnourished metabolic pathology (13, 14).

Fetal period is an important stage when poor environment *in utero* can decide the health destiny of offspring in the whole life (15). Apart from congenital anomalies that are associated with maternal diabetes mellitus and obesity (16, 17), numerous studies on clinical statistics have uncovered the phenomenon that women who experienced intrauterine hyperglycemia were more likely to acquire diabetes mellitus (18) and obesity (19). By comparing the siblings born before and after mothers acquired diabetes mellitus, the healthy condition of sibship differed significantly: after the mothers were diagnosed with diabetes mellitus, babies became vulnerable to acquire a higher BMI (20). In addition, cardiovascular diseases, malformation and dysfunction of organs and systems have aroused great concern among scientists. In this review, we will first discuss the adverse impact that intrauterine hyperglycemia leaves in an offspring (Table 1). Then, the potential mechanisms will be displayed, respectively (Table 2).

**Table 1:** The adverse effects of maternal diabetes on offspring.

**Targets**	**Clinical results**	**Animal experiments**
Pancreas and islets	• Insulin sensitivity and insulin release were reduced in maternal diabetic offspring at 18–27 years old (21). • Fasting insulin in O-T1DM at preschool age was higher compared to GDM and healthy control (22). • No difference in fasting insulin between O-GDM and control offspring (23).	• GTT and GSIS were impaired in maternal diabetes-exposed offspring at adulthood (24). • Insulin secretion, glucose utilization and oxidation were reduced in islets of O-GDM at 15-week-old (25). GSIS worsened when offspring was fed HFHS diet (26). • Insulin and beta cell mass were increased in diabetic fetuses (27). • Fetal pancreas of GDM identified 219 biochemicals with significant changes (28).
Liver	• No difference in IHCL was found in infant of GDM mothers with higher BMI (mean BMI: 24.3 kg/m^2^) (29). • More IHCL was detected in neonates of mothers combined GDM and obese (BMI>30 kg/m^2^) (30). • Adolescent O-T1DM mothers were susceptible to fatty liver (31).	• Hepatic steatosis and triglyceride were more obvious in O-GDM or O-PGDM (32, 33). • Maternal diabetic offspring hepatocytes secreted more pro-inflammatory factors (34, 35). • Liver insulin sensitivity (32, 34, 36, 37) and glucose infusion were impaired in diabetic offspring (38).
Adiposity	• Diabetic-exposed offspring had higher BMI, waist circumference and body fat, increased general and central obesity at 9–11 years old (39). • BMI of O-GDM was 0.89 kg/m^2^ greater than offspring before their mothers got GDM (40) • O-GDM (41) and O-T1DM (42) had more leptin, less adiponectin and FGF21.	• PGDM offspring had greater peripheral fat mass and larger lipid diameter, but less adiponectin (32). • Epididymal adipocytes of PGDM offspring were more insulin-sensitive to glucose uptake (43). • Impaired mitochondrial structure and energy expenditure in BAT of maternal diabetic offspring (44).
Skeletal muscle	• Glucose uptake and TCA cycle flux decreased, mitochondria was fewer in insulin-resistant offspring skeletal muscle of diabetes history (45–47). • O-GDM had decreased *PPARGC1A* in skeletal muscle (48). • Fatty acid flux into myotube and LPL expression were lower in offspring of T2DM parents (49).	• Insulin resistance and deficit in insulin signaling pathway were demonstrated in neonatal skeletal muscle after only 2-day continuous hyperglycemia (50). • Aberrant mitochondrial dynamics and structure were seen in O-PGDM (51).
Cardiovascular system	• CHD (52–57) and fetal diastolic dysfunction (58) were reported in O-PGDM. • Angiogenesis was poor in diabetic HUVECs (59, 60), GDM neonates derived ECFCs (61) and CPCs (62). • Girls of maternal *Diabetes* had higher BP (63) and risks of hypertensive disease, heart failure, deep vein thrombosis and pulmonary embolism as well as risk of early onset (64). • Systolic BP, cardiac output and stroke volume were all higher in O-GDM (65).	• Mitochondrial bioenergetics was disrupted (66), its reserve capacity was poor in male O-GDM cardiomyocytes (67). • PGDM neonatal hearts had inefficient ATP production, increased lipid peroxidation (68), impaired EMT and coronary artery volume (69). • Diastolic function and left ventricular compliance were diminished in GDM male hearts (70). • Diabetic offspring had higher blood pressure (71), greater vasoconstriction (72) and endothelial dysfunction (73) in at adulthood. • O-GDM had bigger ischemia-induced cardiac infarction size (74). • Impairment of artery response, decrease in flow in renal peripheral vessels were found in diabetic offspring (75).
Kidney	• PGDM was associated with strong teratogenic effects on kidney (76) and urinary tract (77). • Maternal glucose level was not associated with fetal kidney volume and dimensions (78). • Urinary calcium and magnesium excretion were lower in offspring aged 5–18 years old of T1DM mothers (79).	• Maternal diabetic offspring showed interstitial fibrosis (80), severe glomerulosclerosis (71, 81), functional glomeruli loss (82, 83), less sodium, calcium and magnesium output (84, 85) and urine volume (86), decreased creatinine clearance (87), increased glomerular filtration rate (88) microalbuminuria (80). • Activation of the intrarenal RAS (80, 82, 88, 89) and dopamine receptor phosphorylation (86) were found in renal cortex of hypertensive offspring of diabetic mothers.
Neuropsychiatry outcomes	• GDM can develop intellectual disability (90, 91) and psychiatric disorders (92, 93) in offspring at a younger age (94), like ASD (95), ADHD (92) and eating disorders (96, 97). • Children of GDM mothers had higher hypothalamic blood flow and sensitivity to glucose stimulation (98).	• Anxiety-like behaviors were reduced (99, 100), recognition memory impairment (101, 102) and short-term memory deficiency (100, 103, 104) were shown in O-GDM. • Brain malformation (105) like disturbed neocortical lamination (106) or neural tube malformation (107) and hippocampal synaptic integrity derangement (101, 108), activated microglia (101) and hippocampal over-excitability (99) were seen in O-GDM.
Reproductive system	• Female offspring of GDM mothers had an earlier onset of puberty (109).	• Female offspring of diabetic mothers showed smaller ovarian section (110), fewer and smaller primary follicles (110, 111), ovulate fewer oocytes after HFD (112). • High glucose increased ovary weight and estrogen levels in diabetic female offspring (113, 114). • Serum testosterone levels and sperm count were decreased (113, 115–117), the anogenital distance was shortened (115) in GDM male offspring. • Anogenital distance index was significantly increased, testes descent and preputial separation were earlier started when exposed to maternal hyperglycemia (118).
Immune system	• The number of lymphocytes went up (119, 120), activating T cells were decreased (119, 121), and the suppressor T cells were increased (120, 122) in offspring of diabetic mothers.	• Male O-PGDM elevated bone marrow myeloid progenitors and total cellularity (123). • Splenocytes secrete more IL1β after GDM offspring fed HFD diet (124). • Neutrophil count fell down (123), the proliferative and chemotaxis ability of circulating lymphocytes were restrained (125, 126), activation of B cells was encouraged (127) in diabetic offspring mesenteric lymph nodes.
Lung	/	• O-GDM had smaller lungs (128), delayed maturation (129) at birth and 3 weeks, increased lung compliance and reduced lung resistance at 10 weeks old, which may be evolved to COPD (130).
Dentification	/	• Maternal diabetes offspring mice had impaired odontogenesis *via* NF-κB signaling (131).

**Table 2:** The potential mechanisms of maternal diabetes act on offspring.

**Mechanisms**		**Clinical results**	**Animal experiments**
Microbiota		• GDM altered the fecal (132–134) microbiota of neonates and infants. • GDM mothers and their children had a similar fecal microbiome composition (135).	• Microbial composition in PGDM mothers can be transmitted to offspring (136). • Co-housing of NOD^low^ and NOD^high^ mothers could increase the diabetic incidence of NOD^low^ offspring (127).
Inflammation		• Innate (120, 123, 137) and acquired immunocytes (119–122) all malfunctioned. • The fecal microbiota of GDM showed inflammatory and immune function (132, 133). • Single-cell atlas of placenta showed the function of immune cells differed between groups (138).	• Balance of pro-inflammatory and anti-inflammatory cytokines (124–126, 139) was broken. • Inflammation inhibited embryo implantation and growth of fetus (140). • Inflammation acts on islets (27, 141), liver (35), adiposity (142), heart (75, 143, 144), kidney (80), brain (101, 145, 146), reproductive system (110, 116, 140), lung (130).
Reactive oxygen species		• Proteomic analysis revealed that proteins involved in redox homeostasis were significantly altered in GDM and associated with increased mitochondrial superoxide generation, protein oxidation, DNA damage, and diminished glutathione synthesis (147).	• Higher level of ROS was detected in heart (68, 69, 74), arteries (148) of GDM offspring. • Oxidative stress was much more in renal vessels of diabetic offspring (75). • Repressed *Sod2* transcription and enhanced ROS accumulation in maternal diabetes-induced neurodegenerative diseases in offspring (100, 107, 149, 150). • Imbalance of oxidant and antioxidant (151) appeared in testicular formation (117) and oocyte-granulosa interaction (111) in diabetic offspring.
Cell viability		• Higher percentage of trophoblast cells exhibited telomere capture in GDM (152). • Telomerase activity was stimulated in cord blood of T1DM and GDM (153). • Telomere length was shortened in peripheral blood of GDM-born girls (154). • DNA damage was observed in adult offspring of T1DM mothers (155).	• Apoptosis and degeneration of cells were stimulated in diabetes-exposed islets (27, 156), cardiomyocytes (68), ECFCs (61) and brains (107, 145). • Enhanced apoptosis and suppressed proliferation in offspring caused subfertility (110, 114, 116) and odontogenesis (131). • Single-cell transcriptomics displayed impeded differentiation in GDM cardiomyocytes (157).
Epigenetics	DNA methylation	• Genome-wide DNA methylation of peripheral leukocytes revealed differentially methylated genes enriched in insulin secretion and pancreatic development (158). • Genes significantly differentially methylated in human GDM placenta and cord blood were mainly enriched in neurological disease and cell death (159, 160). • Hypermethylation of *ADIPOQ* (42) led to insulin resistance in adipose tissue of GDM offspring. • Hypomethylated *DNMT1* was associated with kidney malfunction through genome-wide DNA methylation in GDM adult peripheral blood (161). • Epigenome analysis of umbilical cord of GDM showed alterations mainly in immune system (162), metabolic diseases (121, 163) and ASD (159). • Primary endothelial cells showed differentially methylated genes associated with cellular morphology and movement (164) and apoptosis (165).	• DNA methylation profiles in pancreas displayed differential genes related to development and insulin secretion (166), like *Igf2/H19* (24). • Activation transcription of *Dnmt3a* caused hypermethylation of *Igf2* in high glucose-exposed HepG2 (167). • Differentially methylated genes were associated with insulin resistance in both GDM placenta and neonatal liver (168). • MeDIP and bisulfite sequence showed hypomethylation of *Tnf* (142) in perirenal adiposity of GDM offspring. • DNMT3A caused hypermethylation and down-regulation of *Sirt1* and ischemia-sensitive heart in GDM adult offspring (74). • RRBS of GDM fetal hippocampi showed differentially methylated genes involved in cognitive function (108). • Primordial germ cells of maternal diabetes transmitted methylation status to the next generations (169).
	Histone modification	• Reduced EZH2 and H3K27me3 were found in malfunctioned GDM HUVECs (59). • H3K9ac was downregulated in GDM placentas, which was negatively associated with level of FOXO1 (170).	• Intrauterine hyperglycemia increased P300 and decrease SIRT1, accumulated H3K14ac in the promoter of *Ngn1* and *NeuroD2*, disturbing neural distributions (106). • Hyperglycemia caused H3K9me2 accumulation in *Sod2* promoter, increased ROS and inflammation in hematopoietic stem cells (150). • Reduced H3K27me3 and increased H3K27ac in promoter of *Cartpt* via inhibiting Ezh2 and activating CBP/P300, contributing to GDM subfertility (112).
	MicroRNA	• MiR-15a and miR-15b were increased in skeletal muscle of adult offspring of maternal diabetes (171). • MiR-146a-5p, miR-26a-5p, miR-24a-3p, miR-30a-5p were upregulated in postpartum T1DM plasma exosome (172). • MiR-199a-3p, miR-503-5p, and miR-1268a were increased in GDM amniotic fluid during the second trimester (173). • HUVECs of GDM showed increased miR-101 (59), miR-30c-5p, miR-452-5p, miR-126-3p, miR-130b-3p and miR-148a-3p (174), leading to impaired fat oxidation *via* AMPK signaling. • GDM showed difference of miRNA/mRNA pairs (175), like miR-138-5p and *TBL1X* (176).	• High glucose increased miR-130b-3p expression and secretion, reduced the abundance of *Ppargc1α*, inhibited mitochondrial biogenesis in placental trophoblastic cells (177). • MiR-122 was downregulated in plasma and liver of GDM male fetuses, positively associated with pro-inflammatory status (178). • MiR-139-5p and miR-195-5p were upregulated in fetal heart of PGDM mothers, associating with thickened cardiac wall (179).

## 2. Methods

The National Center for Biotechnology Information (NCBI) search engine (PubMed) was used to extract relevant English-language articles, with keywords such as “Diabet^*^” or “intrauterine hyperglycemi^*^” or “hyperglycemi^*^ in pregnancy” AND “Offspring” together with “islet or pancrea^*^”, “liver or hepatic”, “adipos^*^ or fat or obes^*^”, “skeletal muscle”, “renal or kidney”, “heart or cardiovascular”, “immune or inflammation”, “testis or ovar^*^ or reproducti^*^”, “neural”, and “microbiota” successively. We excluded articles that focused on the influence of paternal diabetes mellitus on offspring. The diet-induced animal model was classified into PGDM. After browsing texts, data extracted from each article included (1) type of research (clinical results or animal experiments), (2) methods for setting up an animal model, (3) subjects in clinical trials (maternal diabetes mellitus type, age, and sex of offspring), and (4) study outcomes/findings.

## 3. The adverse effects of maternal diabetes on offspring

### 3.1. Target on pancreas and islet

Diabetes mellitus is characterized by an insulin deficiency (180) and an insulin resistance (181). Islet consists of β cells, which are the only cell types that can secrete insulin to downregulate blood glucose. A follow-up cohort demonstrated that offspring of GDM and T1DM had reduced insulin sensitivity and insulin release at the age of 18–27 years (21). However, a clinical study reported that offspring of T1DM (O-T1DM) had a higher fasting serum insulin compared to offspring of GDM and heathy control at preschool age (22). No difference in fasting insulin profile was observed between the offspring of GDM (O-GDM) and control mothers in another clinical trial either (23). Due to anatomic position and ethical restrictions, scientists mimic intrauterine hyperglycemia by administration of streptozotocin (24, 115) or alloxan (111, 118) to specifically destroy the islet or feeding high fat high sucrose (HFHS) diet (112, 116) to induce PGDM or GDM in an animal model. Ding, et al. discovered that adult offspring of maternal diabetes presented impaired glucose tolerance and glucose-stimulated insulin secretion (GSIS) in mice (24). Research on rats came up with similar results: 15-week-old GDM offspring rats displayed an impairment in insulin secretion, glucose utilization, and oxidation in islets (25). Their GSIS capacity worsened when O-GDM was fed with a HFHS diet (26). Genes related to the islet development (24), metabolic enzymes (25), insulin secretion, and cell cycle (156) were demonstrated to be downregulated in the islet of Langerhans cells of O-GDM. By infusing glucose into maternal left uterine artery, hyperglycemic-exposed fetuses were observed to have higher levels of serum insulin and a larger β cell mass (27). The metabolomic analysis of fetal pancreas identified 219 biochemicals with significant changes. Among them, citrate acid and α-ketoglutarate were remarkably decreased in O-GDM, which suggested a metabolism-mediated mechanism (28).

### 3.2. Target on liver

Insulin secretion is regulated by pancreatic β cells according to blood glucose fluctuation, while target organs of insulin, including liver, adipose tissue, and skeletal muscle, bind with insulin through an insulin receptor, activate downstream signaling to uptake, and catabolize glucose (182).

As the main metabolic organ of glycogen synthesis, liver has been subjected to extensive research for years. A prospective longitudinal study showed no more intrahepatocellular lipid (IHCL) in infants of GDM mothers with a higher body mass index (BMI, mean BMI: 24.3 kg/m^2^; interquartile range: 21.7, 30.3) compared to control group (29). Another clinical study demonstrated that obese women (BMI>30 kg/m^2^) with GDM gave birth to neonates with more deposition of IHCL (30). In addition, adolescent O-T1DM may predispose to fatty liver (31). In animal models of mice (32) and rats (33), offspring livers of GDM or PGDM presented more detectable steatosis and hepatic triglyceride, accompanied by reduced lipid metabolic enzymes (32). Fat-laden hepatocytes could release hepatokines to induce pro-inflammatory signaling and hepatic insulin resistance (34). Overall, 20-week-old O-GDM mice were actually demonstrated to secrete more pro-inflammatory factors such as interleukin-1β (IL1β), IL6, and IL33 (35). Animal experiments suggested that, even though basal glucose utilization and insulin concentration were normal in O-GDM, glucose infusion into the liver was lower during a hyperinsulinemic clamp (38). This feature was consistent with a decreased level of insulin receptor and dephosphorylation of AKT in O-GDM livers in another study (36), which can be aggravated by postnatal HFHS diet (32). Forkhead Box O **(**FOXO) acts downstream of insulin signaling, which can be activated when insulin signaling is inhibited (182). In the offspring liver of maternal diabetes mellitus, phosphorylation of FOXO was repressed, its enzymatic activity was stimulated, and target genes were upregulated, which represented for a repressed insulin pathway (37).

### 3.3. Target on adipose tissue

A multinational cross-section study conducted in 12 countries showed a higher BMI, waist circumference, and body fat, increased general and central obesity in offspring at the age of 9–11 years once exposed to hyperglycemia *in utero*, but when adjusted for maternal normal weight, the said difference disappeared (39). A prospective cohort study of Swedish men displayed that the BMI of people who experienced intrauterine hyperglycemia was 0.89 kg/m^2^ (95% CI, 0.31 to 1.47) greater than the BMI of siblings born before their mother acquired GDM (40). In animal models, the offspring of PGDM (O-PGDM) had a greater peripheral fat mass and a larger lipid diameter, especially epididymal fat pads. Epididymal adipocytes from O-PGDM were also more responsive to an insulin-stimulated glucose uptake with increased levels of insulin receptor, Acetyl-CoA carboxylase, and glucose transporter 4 (43). The adipose tissue can secrete adipokines, such as leptin and adiponectin, which play a major role in glucose homeostasis and insulin sensitivity. The subcutaneous adipose tissue of O-PGDM was shown to secrete less adiponectin (32). In fact, a Danish follow-up study concluded that O-GDM had a higher leptin, a lower adiponectin, and FGF21 (41). Another clinical observation suggested that the plasma level of leptin increased, while the gene expression of adiponectin in subcutaneous adipose tissue became reduced in the offspring of both GDM and T1DM patients (42).

The brown adipose tissue (BAT) is involved in non-shivering thermogenesis and metabolic homeostasis. However, intrauterine hyperglycemia impaired the BAT mitochondrial structure and restrained its energy expenditure (44).

### 3.4. Target on skeletal muscle

The skeletal muscle accounts for almost half of the lean body mass in an adult and is responsible for the majority of issues related to postprandial and insulin-stimulated glucose disposal. The topic of how the skeletal muscle of an offspring reacts to intrauterine hyperglycemia has remained elusive yet. In insulin-resistant offspring of a T2DM parent or a grandparent, their insulin-stimulated rates of muscle glucose uptake and non-oxidative metabolism decreased as well as a deficit in muscle TCA cycle flux observed (45–47). As mitochondrion is the main organelle for catabolizing fatty acids, less mitochondria and impaired oxidative function can produce a lipotoxic deposit urging insulin insensitivity (183, 184). Insulin-resistant offspring with a family history of diabetes mellitus tend to generate a lower maximal oxygen (O_2_) consumption because of mitochondrial dysfunction (46). Another research has revealed that only O-GDM rather than O-T1DM presented a decreased expression of peroxisome proliferator-activated receptor γ coactivator-1α (*PPARGC1A*) in a skeletal muscle (48). *Ppargc1*α is known to be the key transcription cofactor of mitochondrial biogenesis (185). Another microarray in the offspring of T2DM parents showed that lipoprotein lipase (LPL) was lower, which resulted in a less fatty acid flux into the myotube (49). However, most of the muscle samples collected in these studies were biopsies taken from offspring with a family history of diabetes mellitus rather than from offspring with specific maternal diabetes mellitus, the result of which may be ambiguous. Animal models demonstrated that fetal and neonatal skeletal muscles were insulin resistant after only 2-day continuous intrauterine hyperglycemia (50). Aberrant mitochondrial dynamics and structure were seen in an O-PGDM mice skeletal muscle from F1 to F3 via abnormal oocytes (51).

### 3.5. Target on cardiovascular system

According to retrospective studies, PGDM was reported to be highly associated with congenital heart diseases (CHDs) (52–56) and fetal diastolic dysfunction (58). Even in non-diabetic pregnant women, an elevated glucose level in the first trimester was directly correlated with an increased risk of CHD in the offspring (57). In the animal model, isolated neonatal cardiomyocytes showed disrupted mitochondrial bioenergetics once exposed to late GDM (66), which caused mitochondria-mediated cell death under metabolic stress in aged male offspring (67). PGDM neonatal cardiomyocytes were reported to have cardiac dysfunction and hypertrophy with an inefficient ATP production and an increased lipid peroxidation (68). The fetal heart of PGDM mice exhibited increased reactive oxygen species (ROS) levels, impaired epicardial epithelial-to-mesenchymal transition (EMT), and decreased coronary artery volume (69). Actually, endothelial cells from human umbilical vein endothelial cells (HUVECs) also displayed damaged proliferation and migration and intensive apoptosis in hyperglycemic fetuses (59). A comparative analysis of gene expression showed poor endothelial tube formation and compromised angiogenic capabilities in isolated GDM HUVECs and mesenchymal stem cells from GDM neonates (60). The proteomic analysis of human fetal endothelial cells revealed an alteration in antioxidant signaling in GDM groups. When ROS attacked, nuclear factor erythroid-related factor 2 increased its expression, traveled into nucleus, and bound to an antioxidant response element sequence for restoration of vascular redox homeostasis (147). Endothelial colony-forming cells (ECFCs) play a key role in repairing damaged endothelium and forming new blood vessels. Researchers found enhanced senescence and worsened angiogenesis in GDM neonate-derived ECFCs (61). Another article found that a reduction in circulating progenitor cells (CPCs) in GDM neonates was correlated with loss of angiogenesis (62). Vascular dysfunction could lead to hypertension and heart insufficiency in an adult. It was reported that maternal hyperglycemia in pregnancy was associated with at least one higher blood pressure (BP) value in girls (63), but the difference in BP between groups became insignificant after adjusting for offspring BMI (186). A Danish follow-up study showed that maternal diabetes mellitus was more likely to develop a hypertensive disease, heart failure, deep vein thrombosis, and pulmonary embolism as well as an increased risk of early onset in offspring (64). Furthermore, during the Trier social stress test (TSST), there was an increase in systolic BP, cardiac output, and stroke volume in O-GDM, even after adjusting for various demographic factors such as sex, age, socioeconomic status, and BMI (65). Animal experiments bear witness to the clinical findings mentioned above. Echocardiography demonstrated a significant diastolic dysfunction in O-GDM male rats. In isolated hearts, the baseline cardiac function and left ventricular compliance were significantly diminished (70). Diabetic offspring was also shown to have a higher blood pressure (71), a greater vasoconstriction under electrical stimulation (72), an impaired vascular nitric oxid (NO)–ROS signaling (148), and endothelial dysfunction (73) in adulthood. HFD after birth induced cardiomyocyte hypertrophy, pro-inflammation status, and cardiac trauma in O-GDM (143). Ischemia-induced cardiac infarction size was bigger in O-GDM. Meanwhile, the ROS leveled up, and the heart function was significantly inhibited (74). The impairment of arterial response and a decrease in the flow in renal peripheral vessels were also shown in maternal diabetic offspring (75), which indicated that kidney was another target of intrauterine hyperglycemia.

### 3.6. Target on kidney

As the kidney plays an important role in the pathogenesis of hypertension and diabetic complications, the influence of maternal diabetes mellitus on kidney development in offspring has sprouted out. Epidemiologic studies suggested that O-PGDM was associated with strong teratogenic effects on the kidney (76) and the urinary tract (77). However, another study concluded that the maternal glucose level was not associated with volume and dimensions of fetal kidney (78). When children aged 5–18 years were affected, urinary calcium and magnesium excretion was lower in O-T1DM (79). In animal models, widespread interstitial fibrosis (80) and severe glomerulosclerosis (71, 81) with functional glomeruli loss (82, 83) were present at different periods in maternal diabetic offspring, leading to a decreased creatinine clearance (87), an increased glomerular filtration rate (88), and microalbuminuria (80). Urinary calcium and magnesium output was less (84, 85), similar to the excretion of basal sodium and urine volume (86). Apart from this fact, activation of the intrarenal renin–angiotensin system (RAS), together with intrarenal dopamine receptor phosphorylation (86) and increased transforming growth factor-β1 (*Tgf*β*1*) (80), was evident in the cortex of hypertensive offspring of diabetic mothers (80, 82, 88, 89).

### 3.7. Target on neuropsychiatric outcomes

It also matters whether prenatal exposure to high glucose affects neuropsychiatric development. According to epidemiology, GDM led to adverse neurocognitive and behavioral outcomes in offspring, such as intellectual disability (90, 91) and psychiatric disorders (92, 93). A stratified analysis undertaken on diabetic mothers displayed a higher hazard ratio of autism spectrum disorder (ASD) in their offspring after adjustment (95). A retrospective study suggested that when maternal diabetes required medication, it was positively associated with the incidence of attention deficit/hyperactivity disorder in the offspring (92). Comparing GDM mothers treated with lifestyle or antidiabetic agents with their non-diabetic counterparts, the linear regression analysis showed that O-GDM mothers developed their neuropsychiatric conditions at a younger age (94). Exposure to diabetes mellitus *in utero* also led to eating disorders in the offspring at adolescence (96, 97). The hypothalamic blood flow in response to glucose in children of GDM mothers was obviously higher than control, combined with hypothalamic sensitivity to glucose stimulation (98). Based on experimental research, we arrived at conclusions as follows. First, anxiety-like behaviors were reduced in O-GDM adult mice as seen from the fact that they spent more time in center and open fields than control mice (99, 100). Male mice of GDM were reported to be more alarmed by the fear recall (105); second, impairment of recognition memory was demonstrated by the Novel Object Recognition (NOR) test as the diabetic offspring were not capable of distinguishing familiar from novel objects (101) or after they were fed HFD (102). Additionally, in the radical Y-maze test, O-GDM adult mice made more re-entry mistakes, which indicated a deficiency in short-term memory (100, 103, 104); third, the cell cycle was altered to a stage where apoptosis-related genes were promoted in GDM neonate-derived cells (107). Hippocampi derived from the offspring of diabetic mothers were characterized by cell death and susceptibility to LPS stimulation (145); fourth, hyperglycemia led to malformation of the brain (105), especially disturbed neocortical lamination (106), neural tube malformation (107), and hippocampal synaptic derangement (101, 108). Difference in the number of neurons in the ventromedial nucleus of the hypothalamus vanished after an islet transplantation in the diabetic group (187); fifth, fetal hippocampal formation was exposed to a neuroinflammatory environment because of hyperglycemia, so increased activated microglia and cytokines persisted into young adulthood (101), and O-GDM became more sensitive to an inflammatory response (145). However, another article suggested that microglia were not affected in GDM neonatal hippocampi (104); finally, both hippocampus and cortex were demonstrated to have a higher level of ROS and an enhanced oxidative metabolism together with inhibited superoxide dismutase (SOD) activity (100, 107). Importantly, overexpressing SOD2 in 6-week-old O-GDM can partly rescue its autism-like behavior in 8 weeks (149). There was also some disagreement among researchers on topics such as neural differentiation, where one researcher tested out that neuronal differentiation markers were less in PGDM fetal brains (107); while another stated that neural stem cells isolated from a 7-day-old fetus exited cell cycle in advance and entered premature differentiation after exposed to maternal hyperglycemia (106). In addition, hippocampal excitability was tested in cultured hippocampal neurons: action potentials were larger and the decay time was shorter in hyperglycemic-exposed offspring; and the resting potential was more hyperpolarized because outwards potassium channel density was larger (99).

### 3.8. Target on the reproductive system

As mentioned above, the influence of intrauterine adverse environment could inherit to the second generation (24, 103) through fetal primordial germ cells (169), thus it is reasonable for us to consider that maternal diabetes mellitus leaves negative effects on the reproductive system. Actually, a Danish national cohort study reported that female O-GDM had an earlier onset of puberty (109). In animal models, female offspring of diabetic mothers showed a smaller ovarian section (110). The number and diameter of primary follicles were also decreased (110, 111). O-GDM were more inclined to ovulate fewer oocytes after HFD, thus causing subfertility in adult life (112). Researchers attributed these phenomena to increases in apoptosis (110) and oxidative stress (111). Proteomic analysis showed that differentially expressed proteins identified in ovaries were involved in hydrogen peroxide catabolic process (151). A number of scientists have suggested that, because of lipidomic metabolites (113) and hyperactivity of insulin signaling (114), high glucose increased their ovarian weight and estrogen levels in diabetic female offspring. As for male, serum testosterone levels and sperm count were decreased in GDM male offspring (113, 115–117), possibly due to ROS (117) and apoptosis (116). Researchers once discovered that GDM inhibited glycogen synthase kinase-3β (GSK3β) signaling and delayed the differentiation of stem Leydig cells in fetal testis. Thus, the number of Leydig cells decreased and the anogenital distance was shortened (115). However, in another animal model, scientists suggested that the anogenital distance index was significantly increased, testes descent and preputial separation were started earlier when exposed to maternal hyperglycemia (118).

### 3.9. Target on hemopoietic and immune system

Diabetic mothers were believed to elevate the number of bone marrow myeloid progenitors and total cellularity in male offspring after a 10-month high fat diet (123). Hematopoiesis gives rise to circulating and tissue-resident immune cells (188). In fact, researchers also found that immune cells in offspring failed to function after exposed to maternal hyperglycemia at different periods. Splenocytes were induced to secrete more IL1β after a persistent HFD diet in O-PGDM (124). In the offspring of diabetic mothers, the neutrophil count dropped (123), while the lymphocyte count increased (119, 120). The percentage of activating T cells also decreased (119, 121), so did the capacity of proliferation after antigen stimulation (125, 126). T1DM mothers increased the suppressor T cells in offspring (120, 122). The proliferative ability of B cells was also found to be restrained in diabetic offspring (125). Non-obese diabetic (NOD) mouse colonies can be divided into two groups, NOD^low^ indicates a lower incidence for T1DM development and NOD^high^ indicates a higher incidence for T1DM development (189). B cell activation was demonstrated to be encouraged in mesenteric lymph nodes of NOD^high^ and co-housed NOD^low^ offspring (127). The disturbance of immune system led to higher pro-inflammatory cytokines, such as IL1β, IL6, and tumor necrosis factor-α (TNF-α), and lower anti-inflammatory cytokine such as IL2 (125, 126).

In addition to the organs and systems mentioned above, other organs that are affected by intrauterine hyperglycemia have been researched. Newborns and 3-week-old offspring of diabetic mothers had smaller lungs (128) and delayed maturation (129). Male O-GDM had increased lung compliance and reduced lung resistance at 10 weeks old, which may evolve into a chronic obstructive pulmonary disease (130). A high glucose environment was also demonstrated to activate the apoptosis of primary dental papilla cells and dental epithelial stem cells *via* nuclear factor κB (NF-κB) signaling pathway, resulting in impaired odontogenesis in maternal diabetic offspring mice (131).

## 4. Potential mechanisms

### 4.1. Microbiota

Recently, gut microbiota of mothers was thought to be associated with PGDM and GDM (190–192). Remarkably, the microbiota composition of neonates was associated with GDM pregnancy. One research study stated that GDM was related to increase in microbes that are involved in suppressing an early immune cell function in neonates (132); whereas the other research study suggested that a 7-day-old O-GDM showed a higher relative abundance of pro-inflammatory taxa (133). The fecal microbiota of infants aged 7 days and 9 months showed consistent difference between O-GDM and control, but some taxa did not (134). A cross-section study showed that genus *Anaerotruncus* was increased in 5-year-old children of GDM through gut microbiome. Mothers and their children had a more similar microbiome composition when compared with others (135). The mice model also demonstrated that gut microbiota could be transmitted *via* delivery and perinatal nursing. Multi-omics analysis supported that both glucometabolic deficits and microbial composition in PGDM mothers were subsequently transmitted to their offspring. By Cesarean delivery and cross-fostering, the microbiota vertical transmission and insulin resistance were blocked (136). There was yet another interesting phenomenon. Exposing NOD^low^ mice to NOD^high^ after weaning cannot increase the incidence of T1D. However, if co-housing NOD^low^ and NOD^high^ mothers in advance, the offspring of NOD^low^ acquired increased the incidence of diabetes, which indicated that maternal microbiota and lactation environment were two important transmissible diabetes-promoting factors (127).

### 4.2. Inflammation

Another potential mechanism is inflammation. As we summarized above in the section “Target on hemopoietic and immune system”, immune cells were reprogrammed under intrauterine hyperglycemia. Not only in fetal or maternal cord blood, but also in children's peripheral blood, innate immunocytes such as leukocytes (120, 123, 137) and acquired immunocytes such as lymphocytes (119–122) were all dysfunctional, forming a status of imbalance of increased pro-inflammatory cytokines and decreased anti-inflammatory cytokines (124–126, 139). In periconception diabetic mice, the intrauterine pro-inflammatory factors, such as IL1α, TNFα, and interferon-γ (IFNγ), were increased. Thus, the blastocyst development was impaired, the embryo implantation was inhibited, and the growth and development of the fetus during middle and late gestational periods were delayed (140). Systematic pro-inflammatory cytokines or local immune cells acted on islets (27, 141), liver (35), peripheral adiposity (142), heart (75, 143, 144), kidney (80), brain (145, 146), reproductive tract (140), lung (130), and microbiota (133), resulting in damage to organ function.

### 4.3. Reactive oxygen species

Excess glucose in blood and enhanced intracellular glucose oxidation lead to a mitochondrial overproduction of superoxide (193). The ROS acts as an intracellular toxic substance (194) and amplifies mitochondrial damage of hyperglycemia and lipotoxicity. The role of ROS in the pathogenesis of metabolic diseases (195), including atherosclerosis (196) and neurodegenerative diseases (197), has been highlighted. Diabetes-exposed neonatal cardiomyocytes showed a decreased mitochondrial copy number and an impaired palmitate oxidation. Lipotoxicity (66) and ROS accumulation (69) in the heart resulted in cardiac dysfunction. Impaired NO–ROS signaling and increased superoxide production in GDM-exposed endothelial cells (147) and arteries led to hypertension (148). Furthermore, treatment with antioxidant or mitochondrial transfer in diabetic offspring could rescue deterioration in heart ischemic injury (74) and cardiomyocyte bioenergetics (68). Activation of the oxidative stress pathway was also demonstrated in renal vessels among diabetic offspring (75). SOD2, an enzyme that converts superoxide to less reactive hydrogen peroxide and diffuse freely, is strongly involved in the progression of neurodegenerative diseases (198). The transcription of *Sod2* was found to be repressed through epigenetic mechanisms in maternal diabetes-induced autistic brain (100, 107), including neurons (149) and hematopoietic stem cells (150). In addition, the imbalance in oxidant and antioxidant was suggested to be an underlying factor resulting in testicular malformation (117) and altered oocyte–granulosa interaction (111) in diabetic offspring.

### 4.4. Cell viability

Telomere was thought to be related to cellular lifetime. Higher percentage of trophoblast cells exhibited telomere capture in GDM pregnancies (152). Telomerase activity was higher in cord blood from T1DM and GDM, but not T2DM pregnancies (153). Telomere length was observed to be shortened in GDM-born girls' peripheral blood at 9–16 years of age when compared to the control female. Telomere length showed significant association with maternal GDM status positively and insulin levels or HOMA-IR negatively (154). The absence of telomere shortening and oxidative DNA damage were observed in 16- to 23-year-old adult O-T1DM (155).

Cell cycle was another factor associated with cell viability. Cyclin-dependent kinase (*Cdk*), a cell cycle marker, was transcriptionally inhibited in diabetes-exposed islets (156). Inflammation (145) and ROS (107) in diabetes-exposed brains both could modulate cell cycle, contributing to apoptosis and neurodegeneration. In addition, cell cycle in reproduction plays an important role in gametogenesis. Intrauterine hyperglycemia impaired neonatal folliculogenesis (114) and initiation of meiosis (110) *via* promotion of apoptosis and inhibition of proliferation. *In vivo and in vitro* experiments also demonstrated that hyperglycemia suppressed proliferation and enhanced apoptosis in tooth germs *via* activation of NF-κB signaling pathway (131).

### 4.5. Epigenetics

Gestational complications are thought to influence the offspring by means of epigenetic changes accordingly. Epigenetics mainly consists of DNA methylation, histone modification, and microRNAs without alterations in DNA sequence.

#### 4.5.1. DNA methylation

Genome-wide DNA methylation analysis of peripheral blood revealed the dysregulation of genes related to NOTCH and WNT signaling which were implicated in pancreatic development and insulin secretion (158). Another DNA methylation profile in pancreas of the mouse offspring showed that glucolipid metabolism-related pathways, such as pancreatic secretion and development, were influenced (166). *Igf2* and *H19*, two imprinted genes, were methylated higher and expressed less in islets isolated from the mouse offspring of PGDM mothers (24). Exposed HepG2 cells to a high glucose resulted in many combinations of FOXO1 within DNA methyltransferase 3a (*Dnmt3a*) promoter regions. The activated transcription of *Dnmt3a* caused increased methylation of *Igf2* (167). By overlapping differentially methylated genes in both placenta and neonatal liver, it was believed that GDM could significantly affect the biological function, on the top of which was endocrine disorder such as insulin resistance (168). An article suggested that the subcutaneous adipose tissue was regulated to be hypermethylated and thus downregulated adiponectin expression in GDM offspring, which led to insulin resistance in some sense (42). *Tnf* was hypomethylated and expressed higher in perirenal adiposity of the GDM offspring (142). Hypermethylation of sirtuin 1 (*Sirt1*) via DNMT3A contributed to a lower expression of *Sirt1* and ischemia-sensitive heart in GDM offspring in later life (74). A lower methylated *DNMT1* was also associated with alterations in the genome-wide DNA methylation profile in GDM adult peripheral blood cells, relating to an increased glomerular filtration rate and kidney dysfunction (161). Reduced representation bisulfite sequencing (RRBS) of GDM fetal hippocampi showed that altered methylated genes were involved in cognitive function. Metabolic profiling in this article also identified differential metabolites in fetal brain targeting epigenetic modifications (108).

By collecting clinical placenta and cord blood samples from newborns, researchers found that differentially methylated genes between groups in both tissues concentrated on immunological diseases, metabolic diseases, and endocrine disorders (163). Epigenome-wide and transcriptome-wide clinical analyses displayed that GDM was associated with alterations mainly in the immune system, such as the major histocompatibility complex (MHC) region (162). The umbilical blood of a GDM fetus showed hypomethylation of insulin CpG islands, which may contribute to hyperinsulinemia (121). Meta-analysis of cord blood from GDM newborns displayed lower methylation in olfactory receptor family 2 subfamily L member 13 (*OR2L13*) promoter region (159), which was also reported to be hypomethylated among whole blood cells from ASD patients (199). Another genome-wide DNA methylation profiling of GDM infants showed differentially methylated genes to be mainly enriched in the T1DM pathway, immune MHC-related pathway, neuron development-related pathway, and fetal growth (160). Epigenome-wide association study in cord blood revealed no shared and consistent epigenetic marks between GDM mothers and offspring. However, eight significant CpG sites in *TFCP2, H3C6, LOC127841, UBE3C, and FAM13A* genes were identified to be associated with GDM exposure (200). In addition, genome-wide methylation and transcriptome in primary fetoplacental arterial and vein endothelial cells between groups identified genes to be associated with cellular morphology and movement (164). Placenta-specific 8 (*PLAC8*) was hypomethylated in GDM ECFCs after exposed to intrauterine hyperglycemia, which partly resulted in apoptosis and senescence (165). Additionally, the status of methylation could be transmitted to F2 offspring via F1, whose sperm evolved from F1 primordial germ cells (PGCs) also experienced the same attack in the uterus (169).

#### 4.5.2. Histone modification

Scientists also explained that intrauterine hyperglycemia may increase the expression of P300 and decrease the SIRT1 level in newborn neurons to increase histone H3 on lysine 14 (H3K14) acetylation. H3K14ac increased enrichment in neurogene 1 and neuronal differentiation 2 gene promoter region to activate their transcription to participate in neuronal differentiation, which disturbs the distribution of neocortical neurons (106).

Researchers also found that hyperglycemia led to histone H3 on lysine 9 di-methylation (H3K9me2) combination more in *Sod2* promoter region in autistic mice of maternal diabetes. An increase in oxidative stress and inflammation caused by a lower transcription of *Sod2* inhibited mitochondrial DNA copies and function in hematopoietic stem cells (150). Maternal diabetes also increased miR-101, targeting to capture histone methyltransferase enhancer of zester homolog-2 (*Ezh2*) mRNA and activate histone acetyltransferase. Reduced trimethylation of histone H3 on lysine 27 (H3K27me3) and increased acetylation of histone H3 on lysine 27 (H3K27ac) in CART prepropeptide promoter region induced *Cartpt* expression in subfertility female of diabetic mothers (112). Reduced EZH2 and H3K27me3 were also found in malfunctioning GDM HUVECs (59). Additionally, H3K9ac was downregulated in clinical GDM placentas, which was negatively associated with the level of FOXO1 (170).

#### 4.5.3. MicroRNA

As a hotspot epigenetic modification in the past decade, microRNA was demonstrated to match with single complementary mRNA strand and induce retardation in its translation. From a follow-up study, miR-15a and miR-15b were increased in the skeletal muscle of offspring of a diabetic adult. The correlation analysis showed that the levels of miR-15a and miR-15b were positively associated with OGTT glucose, insulin, and C-peptide levels (171). The HUVECs of GDM and high glucose both displayed increased miR-101, but reduced EZH2b and H3K27me3, thereby associating with decrease in HUVEC functional capacities (59). In another research on HUVECs, GDM displayed higher levels of miR-30c-5p, miR-452-5p, miR-126-3p, miR-130b-3p, and miR-148a-3p, which inhibited the expression of AMP-activated protein kinase-α1 (*AMPK*α*1*) and decreased fatty-acid oxidation (174). In placental trophoblast cells, a high glucose increased miR-130b-3p expression and secretion, which reduced the abundance of *PPARGC1A* and mitochondrial biogenesis (177). miR-146a-5p, miR-26a-5p, miR-24a-3p, and miR-30a-5p were significantly upregulated in plasma exosome-enriched extracellular vesicles from T1DM mothers during the postpartum period (172), probably leading to an immune response at pregnancies. In addition, miR-199a-3p, miR-503-5p, and miR-1268a were increased in GDM amniotic fluid during the second trimester (173). The whole transcriptome profiles in GDM and healthy placenta showed a total of 2817 miRNAs with significant difference (175). GDM placenta showed that downregulated miR-138-5p and its corresponding mRNA, transducin β-like 1 X-linked, were upregulated. The miRNA–mRNA pair was required in proliferation (176). In animal models, the expression of miR-122 was downregulated in plasma and liver of GDM male fetuses and positively associated with pro-inflammatory status (178). The fetal heart of PGDM showed a dramatically thickened cardiac wall and upregulated miR-139-5p and miR-195-5p (179). From the results mentioned above, it seems that miRNA differs according to sex (173) and tissue (201).

## 5. Discussion and prospects

With progress in scientific technologies, transcriptome profiling of human placenta at the single-cell level demonstrated that immunocytes in the GDM placenta played an important role in pathophysiology (138). Single-cell transcriptomics also unveiled embryonic exposure to hyperglycemia perturbed cardiomyocyte differentiation, which might be associated with congenital heart disease (157).

**Figure 1:**
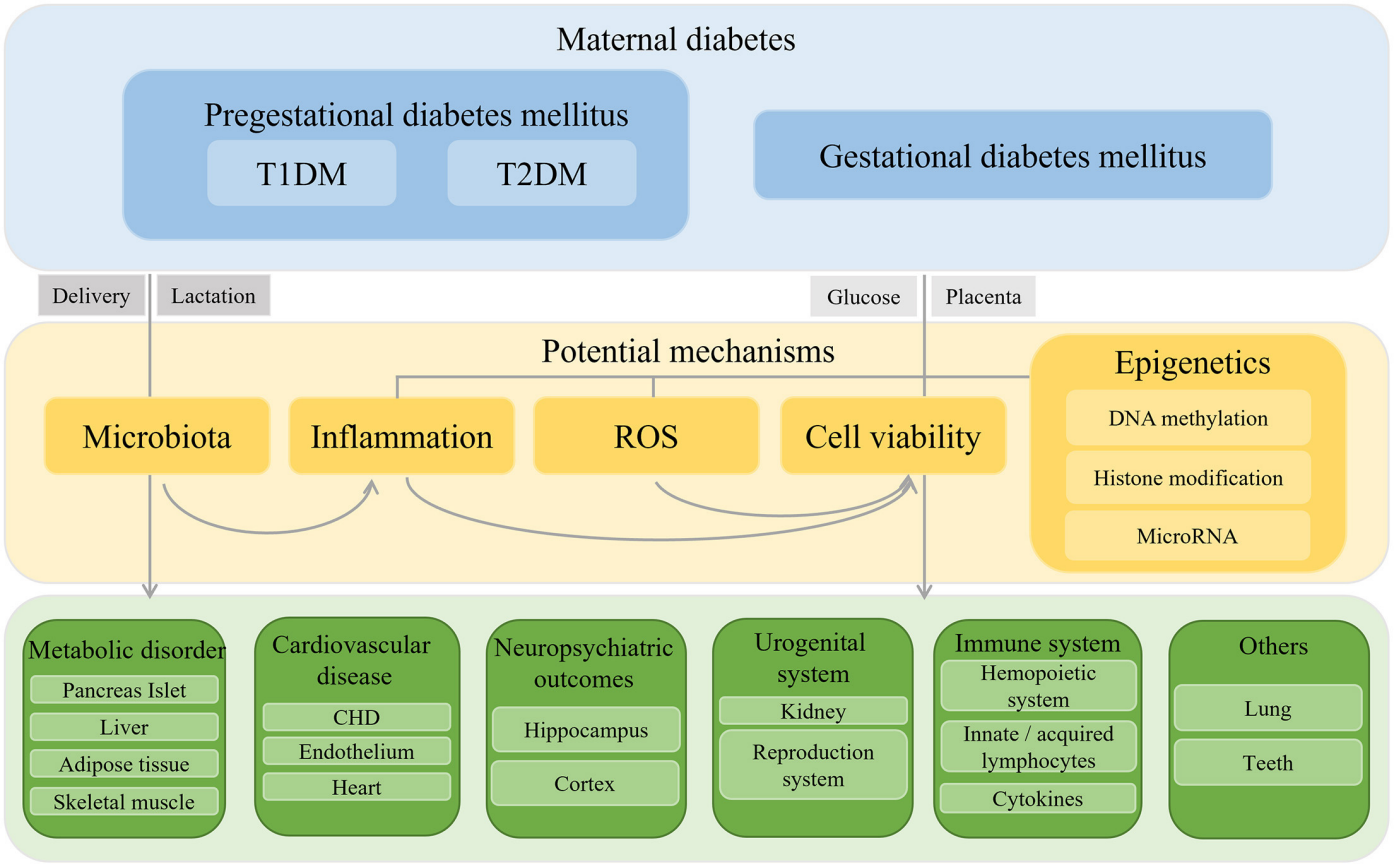
Schematic diaphragm of outcomes and mechanisms of offspring exposed to intrauterine hyperglycemia.

In summary, maternal diabetes creates a hyperglycemic or a hyperinsulinemic environment for the development of the fetus. Glucose or insulin travels across the maternal–fetal barrier and acts on the fetal growth and organic function. Up to now, with clinical data and evidence of animal experiments, researchers have already demonstrated that intrauterine hyperglycemia or hyperinsulinemia caused systematic inflammation in the offspring, disrupted the balance of oxidant and antioxidant leading to an ROS accumulation and DNA damage, altered the expression of vital genes through DNA methylation, histone modification and miRNA, accelerated the apoptosis, and deferred renewal of cells. However, whether maternal glucose or insulin works directly on the offspring or whether there exist another pathogenic molecule, such as miRNA, or cytokines secreted by diabetic placenta, need to be explored. As we can see, different types of maternal diabetes mellitus showed different phenotypes in the offspring but specific mechanism has remained unclear. Regardless of inheritable virulence genes of T1DM or T2DM, the subject of how short-term intrauterine environment works on offspring for a long time is another research point. The affected organs in offspring by intrauterine hyperglycemia present disparate outcomes (Figure 1), meanwhile they influence each other: Dysfunction of islets in offspring exacerbates the glucose level and works on others' functioning. The poor reaction of peripheral insulin effectors lays stress on islets in return. Neural system is another victim of maternal diabetic environment. As a conductor, the dysregulated neural system could play a role in another development. The immune system also acts as both a victim and an initiator in the malformation and dysfunction of offspring. Furthermore, mechanisms we summarized could also work together. An adverse environment alters genetics or epigenetics in the placenta and offspring, bringing about pro-inflammatory gene expression and secretion and mitochondrial dysfunction. Furthermore, gut microbiota in offspring turns into acquiring pro-inflammation status. The ROS accumulation and inflammation result in a cellular inactivity and are short lived. There is no doubt that diabetic mothers provided an unhealthy environment for the fetus, so it is necessary for mothers to keep fit. Our previous study demonstrated that once the epigenetic imprint was left on the offspring by intrauterine hyperglycemia, even with insulin treatment to maintain normal maternal glucose, it is important for the offspring to keep a healthy diet in case of emergence of dysfunction (202). Thus, the question of how to intercept or mitigate the adverse effect of uncontrolled maternal diabetes mellitus is another topic worth emphasis in the future. Scientists suggested that the delivery mode and breast feeding by diabetic mothers can transmit a pro-inflammatory status to the offspring *via* microbiota. As microbiology is now a hotspot, whether there is need to cut intergenerational transmission through C-section and foster nursing remains to be verified. Although existing data can be controversial in some respects, conflicts can arise because of different criteria, classification standard, research method, or animal model. With the increasing occurrence and pronounced influence of gestational diabetes, more attention should be paid to and more effort should be bestowed on the precise mechanism and valid treatment, so that we can guarantee the health of both the mothers and the babies to the maximum extent.

## Author's note

Parts of phenotype have been received by a Chinese journal in Chinese.

## Author contributions

Y-SY wrote the manuscript. CF and MJ wrote the review and editing. ST and YZ prepared the tables. Y-TH and D-QY designed the figure. M-MZ checked the tables. D-QY acquired the funding. Y-TC and JC supervised the manuscript. All the authors approved the final version of the manuscript.
